# Immune compartments at the brain’s borders in health and neurovascular diseases

**DOI:** 10.1007/s00281-023-00992-6

**Published:** 2023-05-03

**Authors:** Jennifer E. Goertz, Lidia Garcia-Bonilla, Costantino ladecola, Josef Anrather

**Affiliations:** 1 Feil Family Brain and Mind Research Institute, Weill Cornell Medicine, 407 East 61St Street; RR-405, New York, NY 10065, USA

**Keywords:** Border-associated immune cells, Brain, Meninges, Choroid plexus, Calvaria, Skull bone marrow, Cirumventricular organs, Stroke, Hypertension, Neuroinflammation

## Abstract

Recent evidence implicates cranial border immune compartments in the meninges, choroid plexus, circumventricular organs, and skull bone marrow in several neuroinflammatory and neoplastic diseases. Their pathogenic importance has also been described for cardiovascular diseases such as hypertension and stroke. In this review, we will examine the cellular composition of these cranial border immune niches, the potential pathways through which they might interact, and the evidence linking them to cardiovascular disease.

## Introduction

The skull and brain feature several distinct border-associated immune compartments that contribute to immune homeostasis and cardiovascular disease. These compartments consist of the cranial bone marrow, dural meninges, leptomeninges, parenchymal perivascular space (Virchow-Robin space), circumventricular organs, choroid plexus, and brain ventricles. They can be considered immune compartments because they are separated from each other by structural and functional barriers that limit the free diffusion of inflammatory mediators and the trafficking of immune cells between each other and the brain parenchyma. Generally, these barriers are established by cell monolayers that are connected via tight junctions (TJs) to limit free diffusion of molecules and cell movement forming the blood-brain (endothelial TJ), CSF-brain (glia limitans/ependymal TJ), blood-CSF (choroid plexus epithelium TJ), dura-CSF (arachnoid TJ), and circumventricular organs (CVO)-CSF (tanycyte TJ) barriers. The structural organization of these barriers has been recently covered by several excellent reviews [1–4], and we will only discuss them as they pertain to immune functions.

## Border-associated immune compartments in brain and skull

### The skull bone marrow

Observations in humans indicate that the diploic space harbors hematopoietic bone marrow in children and young adults but gradually transforms to non-hemopoietic yellow bone marrow during adulthood. Based on postmortem anatomical exanimation and ^59^Fe uptake studies with radiologic evaluation, it has been estimated that 25% of the body’s total hemopoietic active bone marrow is localized in the skull of newborns and progressively declines to 7.6% at the age of 40 years [5]. In mice, the skull bone marrow is primarily confined to regions close to bone sutures and the base of the skull [6, 7]. scRNA-seq studies suggested that the bone marrow of the calvaria contained developing and adult immune cells in a similar composition to the tibial niche and that the immune cells showed comparable transcriptomes [7]. Despite these similarities in cellular composition, some differences in the transcriptomes of immune cells of the calvaria and the tibia were observed. Gene ontology analysis showed downregulation of proliferation genes in hematopoietic stem cells, genes associated with reactive oxygen species production in monocytes and macrophages, and genes related to myeloid cell differentiation in neutrophils when comparing the respective immune cell populations in the skull bone marrow to their counterparts in the tibia [7].

### The dura mater

The dura is one of the most diverse intracranial immune compartments. The immune cells can be divided into resident and transitory, although this distinction is not absolute, and it may depend on immune cell type and disease context.

#### Yolk sac-derived immune cells

Dural border-associated macrophages (BAMs) are tissue-resident macrophages located in close proximity of dural blood vessel [8] and are the most abundant cell type found in the dura [9, 10]. Although they share ontogeny with leptomeningeal and parenchymal macrophages [8], they differ in transcriptomic signatures showing higher MHC class II complex expression than leptomeningeal and parenchymal BAM [10]. MHC class II expression increases with age in mice possibly due to ongoing replacement of dural BAM by bone marrow-derived cells over the lifespan or due to changes in local environmental signals [10].

#### Bone marrow-derived immune cells

Multiple subsets of dendritic cells (DCs) have been detected in the dura [7, 10, 11]. Although conventional DC2 make up the majority of the dendritic cell population in the dura [10, 12], a smaller population of conventional DC1, migratory DC, and plasmacytoid DC are also present [10, 11]. In a study examining the origins of dural DC, adoptive transfer and parabiosis experiments indicated that pre-DCs from the periphery migrate to the meninges where they further differentiate into mature DC [13]. The half-life of dural DC was estimated to be 5–7 days. The results are in contrast with a more recent parabiont study that reported limited recruitment of dural DC from the periphery and implicated that the replenishment occurred primarily from the skull bone marrow and not the circulation [14].

Ly6c^hi^ monocytes and neutrophils are additional myeloid cell populations found in the dura under homeostatic conditions [7, 10, 11]. Dural monocytes and neutrophils seem to be preferentially recruited from the skull bone marrow via transosseous channels and do not maintain the dural pool by local proliferation [14]. The fate of these cells is unclear as they do not use lymphatic conduits to exit the cranium [14].

T cells, including conventional αβ T cells and γδ T cells, NK cells, and innate lymphoid cell type 2 (ILC2) have been described as constituents of the dural immune compartment [10, 11, 15, 16]. Conventional T cells are preferentially localized near dural sinuses, a special organization thought to be important for interaction with antigen presenting cells and lymphatic trafficking. Attraction and retention of T cells in the dural space might be aided by the expression of VCAM1, ICAM1, and Cxcl12 on stromal and endothelial cells [11]. In contrast to dural monocytes, neutrophils, and DC, parabiosis experiments indicate that dural T cells are recruited preferentially from the peripheral blood and not from the skull bone marrow [14]. γδT cells are found in the dura and have been suggested to originate from peripheral sources including the intestine [16]. Similar to T cells, ILC2 are found preferentially around the dural sinus and when compared to lung ILC2, dural ILC2 exhibit lower expression of genes related to inflammation, signal transduction, and metabolism, indicating a more quiescent state [15]. Based on the observation that ILC2 are a long lived tissue-resident immune cell population in other organs, it could be extrapolated that they have a slow turn over with a half-life of more than 4 months in some tissues [17]. This notion is supported by EdU pulse-chase experiments that detected no appreciable drop in ILC2 4 weeks after EdU administration [18].

There is also a prominent population of B cells in the dural meninges that has been found in rodents and primates [19, 20]. Investigations employing immunohistochemistry identified extravascular B cells in the dura, in addition to B cells within the vasculature and lymphatic vessels [21]. These cells tend to preferentially home near the venous sinuses [19, 21]. The studies further investigated the stage of development of these B cells and found dural progenitor populations that were similar in constitution to the ones present in the skull bone marrow. Furthermore, parabiosis experiments indicate that the majority of dural B cell progenitors originate from the skull bone marrow with only partial recruitment from other niches [19] and take advantage of the skull bone marrow-dura channels for transit [21]. Using B cells expressing Mog-specific immunoglobulin chains, it was shown that dural B cells but not B cells of the skull bone marrow or femur were reduced in these mice when compared to wild-type mice, suggesting that the dural niche is important for controlling CNS autoreactive B cells [20]. However, the presence of peripherally educated B cells in the dura has also been reported [22]. Using B cell receptor sequencing, it was found that dural plasma cells predominantly secreted IgA and that their B cell receptor repertoire was similar to that seen in plasma cells of the small intestine, suggesting an intestinal origin of these cells [22].

Besides dural BAM, the other resident meningeal immune cell population predominantly located in the dura of humans and rodents are mast cells [23, 24]. They develop from bone marrow-derived precursors that populate the CNS during development but are found mainly in the ependymal linings of the ventricles and the meninges in the adult where they are recruited from blood precursors [25]. Although the life span of dural mast cells has not been investigated, it is assumed that they can live for weeks to several month based on observations in other tissues [26, 27].

### Subdural meninges and perivascular space

While structurally and anatomically distinct, we will discuss these two compartments largely as a single unit because they are in close communication with each other and, to date, there are no pharmacologic or genetic tools to distinguish immune cell ontogeny and function within these compartments. As in the dura, the major immune cell type are resident macrophages, which are ontogenetically identical to dural macrophages and can be divided into subdural (leptomeningeal) BAM and perivascular macrophages (PVM) by their anatomical location but not by their transcriptomic profiles [28]. Subdural BAM share a similar transcriptomic profile and surface marker expression with MHCII^low^ dural BAM but some differences were also observed including higher *Lyvel* and *P2rx7* expression in subdural BAM [10]. While all PVM express CD206, LYVE-1 expression might be region specific. Immunohistochemical analysis showed that perivascular LYVE-1 immunoreactivity was highest around vessels of the hippocampus, although the study did not address whether this is due to increased expression or increased vascular PVM coverage in this brain region [29]. In the mouse, the parenchymal perivascular space along penetrating arterioles and venules is seeded with PVM after birth, most likely in conjunction with the development and expansion of the perivascular space [28]. These cells have a slow turnover rate, approximating that of microglia [10]. PVM play important roles in regulating paravascular CSF flow, vascular extracellular matrix deposition, and phagocytosis of potentially detrimental proteins [30].

The presence of other immune cells in the subdural meninges under homeostasis is less clear. This is based on the fact that collecting leptomeninges in mice without contamination from adjacent structures (brain, dura) is technically challenging. Moreover, the subarachnoid space under the parietal bone, the most commonly used location for in vivo imaging, extends only 30–80 μm in a healthy mouse which renders it difficult to attribute cells under observation to a specific compartment [31]. Recent developments in whole body tissue clearing combined with light sheet fluorescent microscopy (vDISCO [32]) have the potential to overcome some of these limitations and similar techniques have been applied to study the vascular structure and bone development of the calvarium [33] and the meningeal lymphatic vasculature [34].

### Choroid plexus (ChP)

Like microglia and CNS border-associated macrophages, ChP macrophages in stroma and at the apical side of the ChP epithelium (epiplexus macrophages) are yolk-sack derived and seed the brain early during development [35]. Compared to other brain myeloid cells, stromal ChP macrophages are short-lived (weeks in the adult mouse) and are at least partially replaced by blood monocyte-derived macrophages [36, 37]. In contrast, similar to microglia and PVM, epiplexus macrophages are self-regenerating without contribution of the bone marrow niche [10]. Developmentally the ChP niche is seeded by CD206^high^ (*Mrcl*) macrophages. Over the life span of the mouse CD206^high^ stromal macrophages give way to CD206^low^MHCII^high^ macrophages, although it is unclear if this occurs due to a transcriptional switch in resident cells or if this reflects ongoing replacement by blood monocyte-derived cells [10, 35]. Other cells found in the ChP stroma are lymphocytes, NK cells, monocytes, neutrophils, and DC, although at significantly lower numbers than resident macrophages [10, 35].

### Circumventricular organs

The CVO-brain and CVO-CSF barrier is formed by tanycytes, specialized glial cells that line the third and parts of the fourth ventricle, and specialized astrocytes [38–40]. Given their lack of a structural BBB, macromolecules can freely diffuse into the parenchyma of CVO. Vessel-associated myeloid cells, including possibly PVM, are found in higher numbers in CVO than in other brain regions [41] and are able to sequester blood derived macromolecules [42]. In addition to resident BAM, peripheral immune cells are found in CVO under homeostatic conditions. Reconstitution of Rag2^−/−^lymphopenic mice with GFP^+^ T cells let to robust seeding of the ChP, median eminence (ME), vascular organ of the lamina terminalis (OVLT), and the subfornical organ (SFO) but not the brain parenchyma [43]. T and NK cells have been detected in the ME of mice under homeostatic conditions by scRNA-seq [44]. Similarly, pathology specimens of humans without CNS disorder showed elevated numbers of CD8^+^ T cells in ventral medulla oblongata and area postrema [45].

### CSF and the cranial vascular network as barriers and facilitators of intercompartmental communications

The brain is surrounded by cerebrospinal fluid (CSF) which is produced at high rates (~ 500 ml per day in humans and ~ 140 μl in the mouse) [46] primarily by the ChP epithelium. CSF is drained from the subarachnoid space through the intracranial and spinal venous system but is also collected by intracranial lymphatic vessels or drains along perineural sheets of cranial nerves to reach extracranial and nasal tissues [47, 48]. Under homeostatic conditions, the CSF contains few innate immune cells, while effector and memory T cells constitute the largest population [49]. The migration pattern of these cells has not been fully elucidated. Extravasation of effector T cells from pial vessels and ChP and transarachnoid migration from the dural extracellular space have been observed, and the relative contribution of these pathways might be disease dependent [2, 50]. Under homeostatic conditions, CSF T cells are transitory [49] and egress from the CSF through dural lymphatic vessels to reach deep cervical lymph nodes and through transcribrosal lymphatics to reach the superficial cervical lymph node (LN) [51]. Owing to endothelial fenestrations and lack of TJ, the blood vessel of the dura mater, choroid plexus, and CVO are leaky, resulting in free exchange of macromolecules between the blood and the surrounding tissue. This increased permeability also facilitates the egress of peripheral immune cells into surrounding structures.

Anatomically, the cranial vasculature can be divided into four major districts: pericranial, diploic, meningeal, and cerebral. The majority of the cerebral venous outflow drains into the dural sinuses from where it exits the cranium through the internal jugular vein in humans, while the external jugular vein is the major outflow path in rodents [52, 53]. Therefore, cerebral veins need to transverse the subarachnoid space and penetrate the dura to connect to the draining venous system.

In primates, as in rodents, the diploe between the outer and inner tables of cranial bones is vascularized by an intricate network of thin-walled diploic veins that show formation of lacunar structures. These veins drain mainly to the dural sinuses but connections to the extracranial veins of the scalp by emissary veins are also present [54]. The diploic veins in mice increase both in covered territory and complexity with age [55]. Since the diploic veins, similar to other intracranial veins, are devoid of valves, the blood in these vessels can theoretically flow in both directions, although in a mouse study the flow was directional from the dural layer to the diploic veins [55].

Whereas pia and arachnoid do not have a dedicated blood supply, the dural arterial network is largely provided by branches of the middle meningeal artery, which in rodents originates from the pterygopalatine or stapedial arteries, both of which are branches of the internal or common carotid arteries. By contrast, in primates, the middle meningeal artery arises from the maxillary artery, a branch of the external carotid artery [56]. This could have implications when modeling human disease in rodents.

### Communications between the subarachnoid and dural space

Several studies using intracerebroventricular, lumbar, or cisterna magna injections of tracers have shown functional connections between the subarachnoid space, the dura, and the diploe in rodents and humans [7, 11, 57–59]. However, the anatomical structures underlying these communications are less well defined and therefore debated. Arachnoid granulations are protrusions of villi-like structures of the arachnoid membrane into the dural sinuses and have long been considered as one of the main drainage systems for CSF [60, 61]. They connect with the endothelial layer of the dural sinus that at that point is devoid of the fibrous collagen-rich layers that are characteristic for the dura. Arachnoid granulations can also protrude into parasagittal venous lacunae that receive inputs from diploic and emissary veins [62]. More recently, arachnoid granulations protruding into the diploic space of the calvaria have been described in humans and have been implicated in CSF absorption through contact with diploic veins [63], and a potential role of this efflux pathway for immune signaling in humans has been proposed [58]. Interestingly, arachnoid granulations show marked diversity in location, structure, appearance, and cellular composition and the presence of CD4^+^ T cells and CD11c^+^ myeloid cells in the stroma of some types of arachnoid granulations suggest possible immune function of these structures [64]. Although “bona fide” arachnoid granulations are thought to be absent in rodents, meningeal invaginations into dural venous sinuses that could serve as CSF outflow pathways have been described in mice [65]. CSF could also be transported along arachnoid sheets that protrude into the dural space along penetrating cerebral veins which connect the subdural veins with the dural sinuses [66]. Perivascular routes of tracer dissemination to the dura and diploe after cerebroventricular injections have been described in mice [59].

The brain itself is covered by a continuous layer of astrocyte cell bodies in rodents or by cytoplasmic processes of marginal astrocytes in primates known as the glia limitans, whereas processes of Bergman glia form the glia limitans of the cerebellum [67]. The astrocytes are in close contact with the basal lamina, which is in intimate contact with the pia. This structure forms a tight barrier to the subarachnoid space and limits the exchange of macromolecules and cells between the brain parenchyma and the CSF. The glia limitans extends along penetrating blood vessels, and together with basement membranes and endothelial TJ forms the BBB that renders the blood vessels of the cerebral vasculature impermeable to macromolecules and circulating cells. However, the glia limitans superficialis is largely absent at the olfactory bulbs [67], and together with a discontinuous arachnoid membrane along the cribriform plate [68], the rostral cranial compartment could constitute a site of exchange for macromolecules and possibly immune cells between the brain parenchyma, CSF, and the dura.

### Communication between dural and diploic compartments

There are an estimated 1000 channels in the calvaria of the adult mouse connecting the dura to the bone marrow of the calvaria. They are approximately 80–100 μm in length and have a diameter about 20 μm [69]. Using endothelial and vascular smooth muscle markers, it was established that these channels contain blood vessels [70]. Furthermore, venous tracing studies found that small transosseous vessels connected the dural venous with the diploic venous system possibly by way of these channels [55]. The space surrounding these vessels can carry CSF as shown by the observation that intracisternally injected tracer can be detected in the perivascular space within the bone marrow. Two thirds of imaged channels exhibited perivascular tracer signal, but it is unclear whether CSF can flow through all channels [59]. This paravascular space is also utilized by skull bone marrow myeloid cells to seed the dural space [69].

Collectively, these studies provide support for the existence of anatomically and functionally distinct border-associated immune compartments in the cranium. CVO, ChP, dura mater, and skull bone marrow, while preserving their immunologic identity, are in close communication with circulating immune cells consistent with their lack of a functional BBB. In contrast, the brain ventricles, leptomeninges and parenchymal perivascular spaces are largely shielded from the periphery under homeostatic conditions, and their resident immune cell populations are either long-lived or replenished by autonomous cell replacement similar to the one observed in microglia [36].

## The cranial border-associated immune compartments in aging and disease

### Age-associated changes in cranial immune compartments

Aging is the major non-modifiable risk factor for cardiovascular and cerebrovascular diseases. Age-related changes have been reported across all cranial border-associated immune compartments. B cell progenitors may be less frequent in the aged dural meninges [20]; however, there appears to be an overall increase in total B cells in the dura [21]. This increase is associated with an increased clonal overlap with blood-based B cells and plasma cells, indicating there may be replacement from the periphery over time [21]. In the dura, both total CD4^+^ and CD8^+^ T cells also increase in aging, and their spatial organization changes such that they are primarily localized away from venous sinuses [11]. No age-associated changes have been identified in dendritic cell populations [11]. There is an increase in neutrophils and macrophages in brains of aged mice [71]. These macrophages seem to be involved in an inflammatory feedforward cycle with brain-resident microglia [71]. In monkeys, there is an age-related increase in CD3^+^ T cells in the ChP, meninges, and perivascular spaces of parenchymal vessels [72]. Perivascular T cell infiltrates were primarily located in the white matter, but an increase of parenchymal T cells was also observed. This did not seem to be due to breakdown of the BBB, but it was associated with increased density of inflammatory microglia and cognitive decline [72]. Similar findings were also reported in aged mice where CD3^+^ T cells were predominantly located in perivascular spaces of white matter tracts [73]. Aging could shift immune cell composition of the skull bone marrow in mice resulting in increased cytotoxic T cells and CD11b^high^ B cells [74]. An expansion of dura and ChP resident ILC2 has also been observed in aging and linked to better cognitive performance [18].

### Effects of stroke on cranial immune compartments

#### Skull bone marrow

The cellular composition of the skull bone marrow changes after experimental cerebral ischemia to a higher degree than in other bone marrow compartments. Six hours after focal ischemia produced by transient occlusion of the middle cerebral artery (MCA), the skull marrow contained significantly fewer neutrophils and monocytes than the tibia, possibly reflecting egress of myeloid cells from the skull to the meninges [69].

#### Meninges

**Fig. 1:**
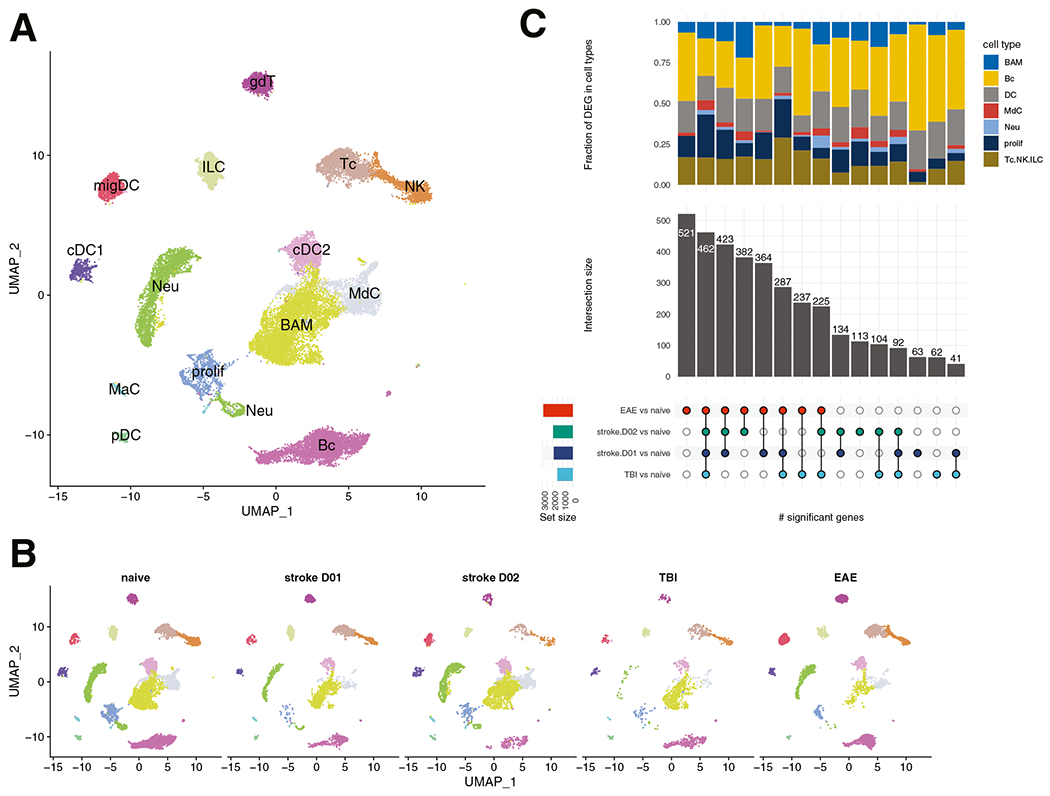
Similarities and differences in the immune response of dural immune cells across different disease models. Single-cell RNA-seq data were compiled from GEO (https://www.ncbi.nlm.nih.gov/geo/) submissions GSE128854 [10], GSE144175 [11], GSE178085 [113], GSE189432 [77], GSE206940 [114], and an unpublished in-house dataset from mice 2 days after transient MCA occlusion. Only samples with libraries from naïve mice, mice undergoing EAE (experimental autoimmune encephalitis), TBI (mild traumatic brain injury), or stroke (transient MCA occlusion) were included. The data was further filtered to select libraries generated from immune cells of the dural meninges from 7 to 16 weeks old mice. **A** UMAP of major immune cell types; border-associated macrophages (BAM), B cells (Bc), conventional DC type 1 (cDC1), conventional DC type 2 (cDC2), γδT cells (gdT), innate lymphoid cells (ILC), mast cells (MaC), monocyte-derived cells (MdC), migratory dendritic cells (migDC), neutrophils (Neu), natural killer cells (NK), plasmacytoid dendritic cells (pDC), proliferating cells (prolif), T cells (Tc). **B** UMAP split by condition; **C)** Upset plot showing overlapping and disease-specific differentially expressed genes (DEG) in response to autoimmunity, mild traumatic brain injury, or stroke

There are also changes to the structure of the meninges after stroke, but the effect could be model dependent. Photothrombotic stroke results in meningeal lymphangiogenesis, a change not observed in transient focal ischemia [75]. This effect appears to be conferred by VEGFR-3, as heterozygous mutant mice had significantly larger infarct volumes after transient ischemia, whereas no difference was observed in photothrombotic stroke [75]. Whether the deleterious effects of VEGFR-3 haploinsufficiency is due to defective lymphatic outflow from the cranium or due to altered systemic lymphangiogenesis and its effect on the peripheral immune system has yet to be determined. Interestingly, acute inhibition of VEGFR-3 signaling by intranasal delivery of a receptor antagonist or lymphad-enectomy of superficial LN in rats decreased brain injury after transient ischemia [76]. Flow cytometry analysis revealed that meningeal B and T cell populations were unchanged at both 24 and 72 h after transient MCA occlusion when compared to mice undergoing sham surgery [77]. In the pia, granulocytes were increased at 24 h and subsequently returned to normal levels by 72 h. There was also a trend that dendritic cells were decreased at both time points, reaching significance in the dura. Dural NK cells were decreased at 24 h and mostly recovered at 72 h, a pattern that was also found in the pia, though without significance. In a mouse model of subarachnoid hemorrhage (SAH), parenchymal perivascular spaces were enlarged and T cells were increased in the meninges 7 days after injury [78]. Gene expression of immune cells in dural meninges is altered by injury and is dependent on the disease model. We reanalyzed publicly available scRNA-seq data from naïve mice and mice after ischemic stroke, mild traumatic brain injury, or experimental autoimmune encephalomyelitis (EAE). This analysis showed while the overall qualitative composition of immune cell clusters found in the dura was not changed, there were overlapping and disease-specific changes in the transcriptome (Fig. 1). EAE showed the highest number of uniquely differential regulated genes when compared to naïve animals whereas the majority of differential regulated genes in the other disease models were shared across conditions and only few were disease specific (Fig. 1C). This may reflect the different pathoetiology of EAE which is mainly characterized by an adaptive immune response while stroke and traumatic brain injury are, at least in the acute phase, driven by innate immunity.

Suggestive of a local expansion of BAM after ischemic brain injury, a histological investigation in rats found an increase in cells expressing the BAM marker CD163^+^, in the leptomeninges 3 days after transient MCA occlusion [79]. These cells were also positive for the proliferation marker Ki67 [79]. Another study in rats also examining CD163^+^ cells suggested this increase began not earlier than 24 h after ischemia [80]. Similarly, using CD206 as a BAM marker, it was found that BAM increased in meninges and the perivascular space along penetrating vessels from day 1 to 3 after transient MCA occlusion in rats; however the study suggested migration from the meninges as the main mechanism for this increase [81]. Interestingly, a study of six human cases with a history of MCA infarction found increased MHC class II positive PVM in the spinal cord weeks to several years after the ischemic event. The PVM were also rich in lipid droplets indicating that these macrophages might be involved in the pyramidal tract degeneration observed after ischemic brain injury [82]. Whether these cells contribute to Wallerian degeneration or constitute a reactive response remains to be established, but the study suggests that after stroke, PVM could play a role in neurodegeneration or repair far away from the site of primary injury.

#### Choroid plexus

The ChP has also been identified as a target organ after ischemic and hemorrhagic stroke [83]. Focal ischemia in rats resulted in a proliferative response as early as 2 h after injury, although immune cells were not investigated in this study [84]. In addition, intraluminal MCA occlusion models might decrease blood flow in the lateral ventricle ChP, produce ChP edema [85] and induce cell death in the ipsilateral ChP [86]. Using a model of distal MCA occlusion in mice, it was shown that expression of *Vcaml, Cx3cl1, Madcaml*, and *Nt5e* (CD73) was increased after stroke [87], indicating that activation of the ChP can also occur in stroke models that do not produce a lesion close to the ChP such as the intraluminal filament model. Using flow cytometry it was shown that monocyte-derived cells increased in the ChP 24 and 72 h after stroke whereas NK cells were reduced [77]. In a model of neonatal stroke, monocytes and neutrophils were recruited to the ChP as early as 3 h after injury and leukocyte infiltration was partially impaired in mice lacking the scavenger receptor CD36 [88].

### Involvement of cranial border immune compartments in stroke

#### Border-associated macrophages

The role of BAM in different stroke models has been mainly addressed by depleting BAM with intracerebroventricularly delivered clodronate liposomes, which are selectively taken up by BAM resulting in apoptotic cell death [89, 90]. A major limitation of this approach is that BAM are depleted in all meningeal and ChP compartments, making it impossible to attribute observed effects to a specific immune niche. Moreover, effects of clodronate liposomes on other immune cells of the cranium, including dural monocytes/neutrophils and those of the skull bone marrow, cannot be excluded, especially under disease conditions when the phagocytic activity of myeloid cells might be increased.

When BAM were depleted 4 days before ischemia/reperfusion in rats, there was no difference in infarct volume, and there were no significant differences in parenchymal infiltration of T cells, NK cells, or monocytes while recruitment of neutrophils to the ischemic cortex, and disruption of the BBB was diminished [80]. In a study of SAH, clodronate treatment 3 h after SAH induction resulted in reduced sensorimotor deficits, but had no effect when BAM were depleted before SAH induction [91], possibly indicating a protective effect of BAM during the hyper-acute phase of injury or effects of clodronate on peripheral monocytes infiltrating the site of injury. Neutrophils primarily seed the dura after SAH while monocytes and T cells were found in the parenchyma. The infiltration of neutrophils and cognitive impairment after SAH was dependent on their expression of myeloperoxidase, whereas deletion of the essential p47-phox subunit of the NADPH oxidase complex, a major source of radicals, or deletion of neutrophil elastase had no effect [92]. Adding additional complexity, the roles of BAM in stroke might be context dependent. In a model of chronic alcohol exposure in mice, which induces microglia and endothelial inflammatory priming, it was found that depletion of BAM prevented the aggravating effect of alcohol on ischemic brain injury while it had no effects in stroke mice not exposed to alcohol [93].

#### Dural mast cells

Because mast cells contain granules with vasoactive molecules and proteases, they have been implicated in BBB disruption and in promoting neutrophil extravasation in the course of cerebral ischemia [24, 94, 95]. Using c-kit mutant mice that show mast cells deficiency, it was shown that depletion of mast cells resulted in decreased levels of neuroinflammation, brain edema, neutrophil infiltration, and infarct size following stroke [95, 96]. Repopulating meningeal mast cells by transfer of in vitro generated bone marrow-derived mast cells reverted the protective phenotype in c-kit mutant mice, and this was partially dependent upon the ability of mast cells to produce IL-6 [95]. Similarly, preventing mast cell degranulation by intracerebroventricular delivery of cromoglycate reduced brain edema and neutrophil infiltration both in ischemic and hemorrhagic stroke models [94, 96, 97].

#### T cells

Dural T cells are continuously recruited from the periphery [14] including the small intestine [16, 98]. Whether the local dural environment affects their phenotype has not been investigated, but there seems to be retention of the activation state imprinted in the periphery. For example, while the number of total dural γδT was not changed in mice with altered microbiota after stroke, they produced more IL-17, which was mirrored by increased IL-17 production in γδT cells in the small intestine. While γδT cells were not found in the brain parenchyma during the acute phase of ischemic brain injury, the study suggested that IL-17 produced in the dura could affect stroke outcome by enhancing parenchymal neutrophil recruitment [16].

#### The roles of leptomeninges and ChP in the immune cell recruitment after stroke

Several studies have addressed the role of leptomeninges as a source of blood-borne immune cells after stroke [77, 99–103]. Consistent with a meningeal origin, neutrophils are found on the abluminal site of leptomeningeal vessel within hours after stroke in permanent and transient ischemia models in rodents. A strong association of neutrophils with leptomeningeal vessel has also been observed in tissue samples from human stroke victims [101]. Whether neutrophils that extravasated in the subarachnoid and perivascular space go on to infiltrate the ischemic territory remains to be established, but the fact that accumulation in the leptomeninges precedes the appearance of neutrophils in the brain parenchyma supports such a scenario [99–101]. The leptomeninges showed accumulation of macrophages and neutrophils within 1 day after permanent distal MCA occlusion in spontaneously hypertensive rats, suggestive of a meningeal role in the immune cell infiltration after stroke [102]. In support of these findings, a study in mice specifically addressing the distribution of neutrophils between different cranial immune compartments after transient focal ischemia found that neutrophils were increased in the leptomeninges and perivascular spaces early after ischemia preceding their accumulation in the brain parenchyma [103]. In contrast to leptomeninges, dural neutrophils did not change after stroke [77].

The ChP is also involved in the recruitment of monocytes and T cells after stroke. Using cell tracking in mice, monocyte-derived cells were found in the ChP and CSF one day after focal ischemia [87]. The number of cells in the CSF was decreased at day 3 after stroke while the number of labeled monocyte-derived cells in the ischemic territory was increased at this time point, suggesting a ChP-CSF-brain route for the recruitment of these cells [87]. Photothrombotic lesioning of ChP reduced T cell infiltration into the ipsilateral cortex after permanent distal MCA occlusion in mice [86]. Interestingly, blocking of CSF flow by Matrigel injection into the ipsilateral ventricle did not significantly alter T cell counts in the ischemic hemisphere, indicating that migration via the ventricular space might not constitute the main route for T cell recruitment to the ischemic territory. It was proposed that direct parenchymal infiltration from the ChP into adjacent brain structures and dissemination along callosal white matter tracts allows T cells to reach the peri-infarct regions [86].

The evidence presented above underlines the complex roles of border-associated immune compartments in ischemic brain injury, and while immune cells of those compartments seem not to be involved in promoting tissue injury, they might play an important role in orchestrating the inflammatory response of brain resident and infiltrating immune cells.

### Cerebral amyloid angiopathy (CAA)

Due to their perivascular localization and phagocytic capacity, PVM are particularly suited to control the flux and participate in the clearance of potentially harmful molecules such as Aβ aggregates. BAM depletion in an early-onset mouse model of Alzheimer’s disease (AD) with parenchymal Aβ plaques and CAA like pathology (TgCRND8 mice), increased perivascular Aβ deposits one month after intracerebroventricular clodronate administration suggest that BAM are involved in vascular Aβ clearance [104]. In contrast, BAM might contribute to neurovascular dysfunction and cognitive impairment in a late-onset mouse AD model (Tg2576). Depleting BAM at 3 months of age, when there was neurovascular impairment but no Aβ plaques, resulted in reversal of neurovascular dysfunction. The deleterious effects of BAM in this model were linked to increased oxidative stress induced by Aβ signaling through the scavenger receptor CD36 resulting in NADPH oxidase activation, reactive oxygen species production, and cerebrovascular dysfunction [105].

Taken together, these studies show that the contribution of BAM to cerebrovascular pathologies found in AD might be disease stage dependent, being a driver of cerebrovascular dysfunction during the prodromal stages while contributing to Aβ clearance once intramural Aβ accumulation has occurred.

### Systemic cardiovascular diseases

Endothelial dysfunction has a major impact on the bone marrow hematopoietic niche, as demonstrated in mouse models of hypertension, atherosclerosis, and myocardial infarction [106]. In the skull bone marrow of mice with hypertension, the study found increased angiogenesis, perivascular collagen deposition, and integrin expression resulting in augmented leakiness and endothelial dysfunction. These changes in the vascular niche were associated with increased hematopoietic progenitor proliferation and higher numbers of circulating innate immune cells [106]. Hypertension might also induce shifts in intracranial immune cell distribution. For example, middle-aged spontaneous hypertensive rats (SHR) showed accumulation of intravascular NK and T cells in the microvasculature of the brain parenchyma when compared to normotensive rats in which most NK and T cells are found in the ChP and meninges. These changes were correlated with increased endothelial VCAM1 and decreased P-selectin expression [107]. SH rats also showed increased numbers of PVM in the brain parenchyma [108]. Elimination of BAM by intracerebroventricular injection of clodronate liposomes diminished neuronal activity in the periventricular nucleus of the hypothalamus and the rostral ventrolateral medulla, two brain nuclei involved in blood pressure regulation, and decreased the blood pressure elevation observed in SH rats between 8 and 10 weeks of age without affecting peripheral inflammation as reflected by circulating Il-1β levels [108]. The study also found that the blood pressure and sympathetic tone increase after systemic IL-1β administration was attenuated in rats with intracranial BAM depletion, indicating that hypertension upon peripheral inflammation might depend on BAM to relay systemic signals to cardiovascular centers in the brain. Similarly, the sympathetic activation and blood pressure increase observed after intracarotid TNF injection was abrogated in normal rats treated with intracerebroventricularly delivered clodronate liposomes. It was suggested that this response was dependent on cyclooxygense-2 mediated PGE_2_ production in PVM of the paraventricular nucleus which increased neuronal activity leading to an elevated sympathetic tone [109].

This is in contrast with studies in spontaneously hypertensive mice (BPH/J2) and mice with slow pressor Ang II hypertension where clodronate BAM depletion had no effect on systemic blood pressure but ameliorated BBB dysfunction and improved cognitive performance [110, 111]. This effect was partially dependent on the presence of angiotensin type 1A receptors and NADPH oxidase activity in BAM.

Salt-induced hypertension results in neurovascular dysfunction and cognitive impairment [110]. A recent study identified dural IL-17-producing γδ T cells and PVM as major drivers of the dysfunction observed in this model. It was shown that intracranial IL-17 production by dural T cells triggered increased superoxide production in PVM through IL-17RA signaling. Because superoxide is highly reactive with nitric oxide, a major endothelium-derived vasodilator, this resulted in decreased nitric oxide bioavailability leading to impaired neurovascular coupling and cognitive performance without affecting systemic arterial blood pressure [112].

## Conclusions and future directions

**Fig. 2:**
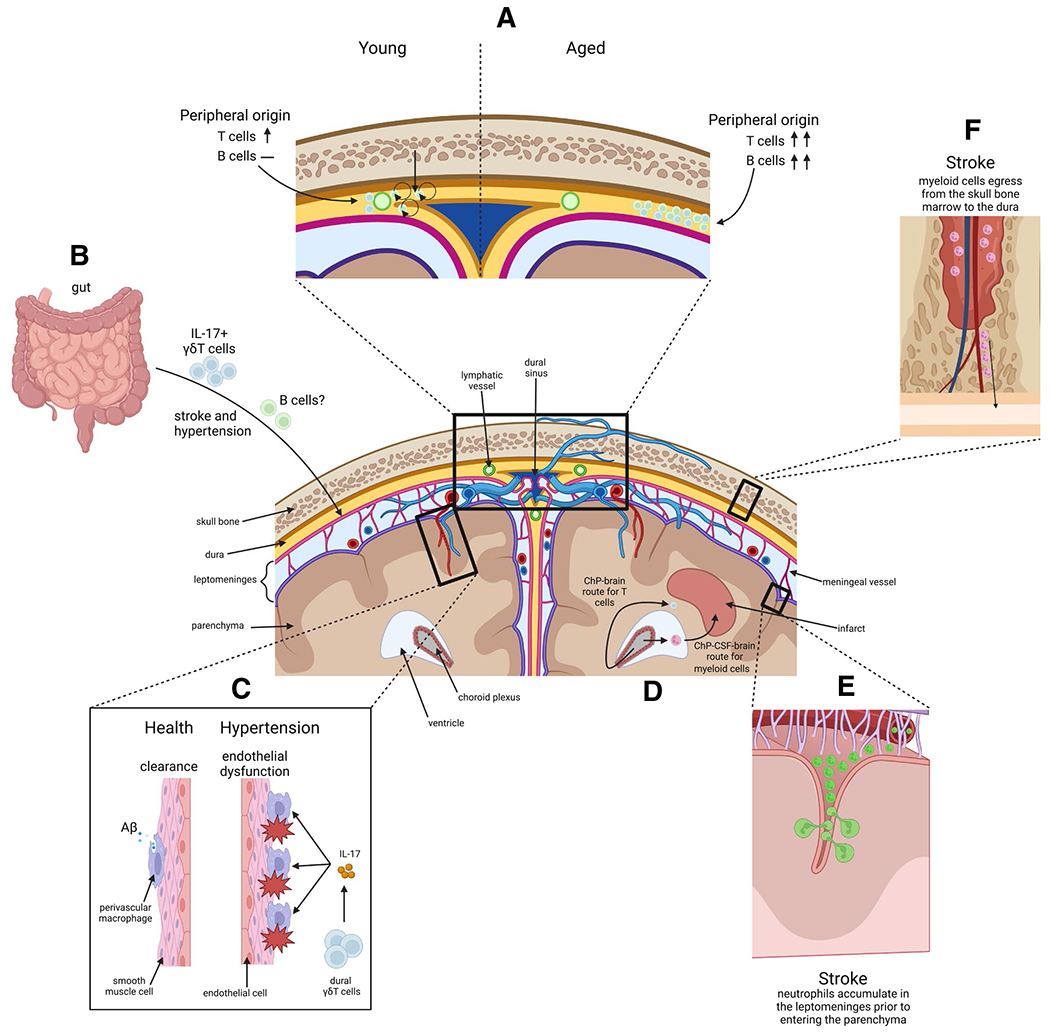
Effects of age and cerebrovascular disease on border immune compartments of the cranium. **A** In young mice, self-proliferating B cells originating from the skull bone marrow and T cells originating from the periphery organize around the lymphatic vessels and sinuses in the dura. In aged mice, these cells originate only from the periphery and are increased in abundance, organizing away from dural sinuses. **B** Some of the dural B cells might be entrained in the gut and IL-17 producing γδT cells migrate from the gut to the meninges following stroke or hypertension. **C** Perivascular macrophages line penetrating vessels of the brain. In health, these macrophages are beneficial in clearing debris and facilitating paravascular fluid movement. In hypertension, they can promote endothelial dysfunction through ROS production. **D** Myeloid cells follow a ChP-CSF-brain route to infiltrate the infarct, whereas T cells enter the brain parenchyma adjacent to the choroid plexus to reach the peri-infarct region. **E** Neutrophils accumulate in the leptomeninges following stroke before they extravasate into the parenchyma. **F** After stroke, myeloid cells egress from the skull bone marrow into the dura

The border-associated immune compartments of the brain, its coverings, and the skull are embedded in structures that allow molecular and cellular communication between themselves and the systemic circulation (Fig. 2). Although their roles in the pathophysiology of cardiovascular diseases are increasingly appreciated, several central questions remain to be addressed. Assuming that the localization to intracranial immune compartments is not stochastic, what are the signals for immune cell recruitment and retention to CVO, ChP, and dura? What is the impact of ongoing replacement of resident immune cells by blood-derived cells during aging on the function of these compartments and their roles in cardiovascular disease? How are signals from these border-associated immune compartments transmitted to the brain? What is the effect of cardiovascular disease and co-morbidities on the composition, function, and reactivity of these immune niches? Answering some of these questions will provide new insights into the function of border-associated immune compartments of the cranium and provide the conceptual framework for the development of therapeutic approaches to target these immune niches.

## References

[1] Alves de Lima K, Rustenhoven J, Kipnis J (2020) Meningeal immunity and its function in maintenance of the central nervous system in health and disease. Ann Rev Immunol 38:597–620 32340575 10.1146/annurev-immunol-102319-103410

[2] Mastorakos P, McGavern D (2019) The anatomy and immunology of vasculature in the central nervous system. Sci Immunol 4(37):eaav0492 31300479 10.1126/sciimmunol.aav0492 PMC6816468

[3] Engelhardt B, Vajkoczy P, Weller RO (2017) The movers and shapers in immune privilege of the CNS. Nat Immunol 18(2):123–131 28092374 10.1038/ni.3666

[4] Coles JA (2017) Where are we? The anatomy of the murine cortical meninges revisited for intravital imaging, immunology, and clearance of waste from the brain. Prog Neurobiol 156:107–148 28552391 10.1016/j.pneurobio.2017.05.002

[5] Cristy M (1981) Active bone-marrow distribution as a function of age in humans. Phys Med Biol 26(3):389–400 7243876 10.1088/0031-9155/26/3/003

[6] Kosaras B (2009) Sensory innervation of the calvarial bones of the mouse. J Comp Neurol 515(3):331—348 19425099 10.1002/cne.22049 PMC2710390

[7] Mazzitelli JA (2022) Cerebrospinal fluid regulates skull bone marrow niches via direct access through dural channels. Nat Neurosci 25(5):555–560 35301477 10.1038/s41593-022-01029-1 PMC9081158

[8] Sato T (2021) Morphology, localization, and postnatal development of dural macrophages. Cell Tissue Res 384(1):49–58 33433687 10.1007/s00441-020-03346-y

[9] Mrdjen D (2018) High-dimensional single-cell mapping of central nervous system immune cells reveals distinct myeloid subsets in health, aging, and disease. Immunity 48(2):380–395.e6 29426702 10.1016/j.immuni.2018.01.011

[10] Van Hove H (2019) A single-cell atlas of mouse brain macrophages reveals unique transcriptional identities shaped by ontogeny and tissue environment. Nat Neurosci 22(6):1021–1035 31061494 10.1038/s41593-019-0393-4

[11] Rustenhoven J (2021) Functional characterization of the dural sinuses as a neuroimmune interface. Cell 184(4):1000–1016.e27 33508229 10.1016/j.cell.2020.12.040 PMC8487654

[12] Mundt S (2019) Conventional DCs sample and present myelin antigens in the healthy CNS and allow parenchymal T cell entry to initiate neuroinflammation. Sci Immunol 4(31):eaau8380 30679199 10.1126/sciimmunol.aau8380

[13] Anandasabapathy N (2011) Flt3L controls the development of radiosensitive dendritic cells in the meninges and choroid plexus of the steady-state mouse brain. J Exp Med 208(8):1695–1705 21788405 10.1084/jem.20102657 PMC3149213

[14] Cugurra A (2021) Skull and vertebral bone marrow are myeloid cell reservoirs for the meninges and CNS parenchyma. Science 373(6553):eabf7844 34083447 10.1126/science.abf7844 PMC8863069

[15] Gadani SP (2016) Characterization of meningeal type 2 innate lymphocytes and their response to CNS injury. J Exp Med 214(2):285–296 27994070 10.1084/jem.20161982 PMC5294864

[16] Benakis C (2016) Commensal microbiota affects ischemic stroke outcome by regulating intestinal gammadelta T cells. Nat Med 22(5):516–523 27019327 10.1038/nm.4068 PMC4860105

[17] Schneider C (2019) Tissue-resident group 2 innate lymphoid cells differentiate by layered ontogeny and in situ perinatal priming. Immunity 50(6):1425–1438.e5 31128962 10.1016/j.immuni.2019.04.019 PMC6645687

[18] Fung ITH (2020) Activation of group 2 innate lymphoid cells alleviates aging-associated cognitive decline. J Exp Med 217(4):e20190915 32022838 10.1084/jem.20190915 PMC7144523

[19] Schafflick D (2021) Single-cell profiling of CNS border compartment leukocytes reveals that B cells and their progenitors reside in non-diseased meninges. Nat Neurosci 24(9):1225–1234 34253922 10.1038/s41593-021-00880-y

[20] Wang Y (2021) Early developing B cells undergo negative selection by central nervous system-specific antigens in the meninges. Immunity 54(12):2784–2794.e6 34626548 10.1016/j.immuni.2021.09.016

[21] Brioschi S (2021) Heterogeneity of meningeal B cells reveals a lymphopoietic niche at the CNS borders. Science 373(6553):eabf9277 34083450 10.1126/science.abf9277 PMC8448524

[22] Fitzpatrick Z (2020) Gut-educated IgA plasma cells defend the meningeal venous sinuses. Nature 587(7834):472–476 33149302 10.1038/s41586-020-2886-4 PMC7748383

[23] Dimlich RV (1991) Linear arrays of homogeneous mast cells in the dura mater of the rat. J Neurocytol 20(6):485–503 1869885 10.1007/BF01252276

[24] Lindsberg PJ, Strbian D, Karjalainen-Lindsberg M-L (2010) Mast cells as early responders in the regulation of acute blood-brain barrier changes after cerebral ischemia and hemorrhage. J Cereb Blood Flow Metab 30(4):689–702 20087366 10.1038/jcbfm.2009.282 PMC2949160

[25] Silver R, Curley JP (2013) Mast cells on the mind: new insights and opportunities. Trends Neurosci 36(9):513–521 23845731 10.1016/j.tins.2013.06.001

[26] Padawer J (1974) Mast cells: extended lifespan and lack of granule turnover under normal in vivo conditions. Exp Mol Pathol 20(2):269–280 4363128 10.1016/0014-4800(74)90059-8

[27] Kiernan JA (1979) Production and life span of cutaneous mast cells in young rats. J Anat 128(Pt 2):225–238 438085 PMC1232929

[28] Masuda T (2022) Specification of CNS macrophage subsets occurs postnatally in defined niches. Nature 604(7907):740–748 35444273 10.1038/s41586-022-04596-2

[29] Karam M (2022) Heterogeneity and developmental dynamics of LYVE-1 perivascular macrophages distribution in the mouse brain. J Cereb Blood Flow Metab 42(10):1797–1812 35751367 10.1177/0271678X221101643 PMC9536125

[30] Drieu A (2022) Parenchymal border macrophages regulate the flow dynamics of the cerebrospinal fluid. Nature 611(7936):585–593 36352225 10.1038/s41586-022-05397-3 PMC9899827

[31] Marchetti L, Engelhardt B (2020) Immune cell trafficking across the blood-brain barrier in the absence and presence of neuroinflammation. Vasc Biol 2(1):H1–H18 32923970 10.1530/VB-19-0033 PMC7439848

[32] Cai R (2019) Panoptic imaging of transparent mice reveals whole-body neuronal projections and skull-meninges connections. Nat Neurosci 22(2):317–327 30598527 10.1038/s41593-018-0301-3 PMC6494982

[33] Rindone AN (2021) Quantitative 3D imaging of the cranial microvascular environment at single-cell resolution. Nat Commun 12(1):6219 34711819 10.1038/s41467-021-26455-w PMC8553857

[34] Jacob L (2022) 3D-imaging reveals conserved cerebrospinal fluid drainage via meningeal lymphatic vasculature in mice and humans. bioRxiv. 10.1101/2022.01.13.476230

[35] Dani N (2021) A cellular and spatial map of the choroid plexus across brain ventricles and ages. Cell 184(11):3056–3074.e21 33932339 10.1016/j.cell.2021.04.003 PMC8214809

[36] Goldmann T (2016) Origin, fate and dynamics of macrophages at central nervous system interfaces. Nat Immunol 17(7):797–805 27135602 10.1038/ni.3423 PMC4968048

[37] Cui J, Xu H, Lehtinen MK (2021) Macrophages on the margin: choroid plexus immune responses. Trends Neurosci 44(11):864–875 34312005 10.1016/j.tins.2021.07.002 PMC8551004

[38] Langlet F (2013) Tanycyte-like cells form a blood-cerebrospinal fluid barrier in the circumventricular organs of the mouse brain. J Comp Neurol 521(15):3389–3405 23649873 10.1002/cne.23355 PMC3973970

[39] Morita S (2016) Heterogeneous vascular permeability and alternative diffusion barrier in sensory circumventricular organs of adult mouse brain. Cell Tissue Res 363(2):497–511 26048259 10.1007/s00441-015-2207-7

[40] Garcia-Caceres C (2019) Role of astrocytes, microglia, and tanycytes in brain control of systemic metabolism. Nat Neurosci 22(1):7–14 30531847 10.1038/s41593-018-0286-y

[41] Takagi S (2019) Microglia are continuously activated in the circumventricular organs of mouse brain. J Neuroimmunol 331:74–86 29107327 10.1016/j.jneuroim.2017.10.008

[42] Willis CL, Garwood CJ, Ray DE (2007) A size selective vascular barrier in the rat area postrema formed by perivascular macrophages and the extracellular matrix. Neuroscience 150(2):498–509 17945430 10.1016/j.neuroscience.2007.09.023

[43] Song C (2016) Expansion of brain T cells in homeostatic conditions in lymphopenic Rag2−/−mice. Brain Behav Immun 57:161–172 27013354 10.1016/j.bbi.2016.03.017 PMC5010944

[44] Chen ZH (2022) Single-cell transcriptomic profiling of the hypothalamic median eminence during aging. J Genet Genomics 49(6):523–536 35032691 10.1016/j.jgg.2022.01.001

[45] Loeffler C (2011) Immune surveillance of the normal human CNS takes place in dependence of the locoregional blood-brain barrier configuration and is mainly performed by CD3(+)/CD8(+) lymphocytes. Neuropathology 31(3):230–238 21092063 10.1111/j.1440-1789.2010.01167.x

[46] Liu G (2020) Direct Measurement of Cerebrospinal Fluid Production in Mice. Cell Rep 33(12):108524 33357428 10.1016/j.celrep.2020.108524 PMC8186543

[47] Louveau A (2015) Structural and functional features of central nervous system lymphatics. Nature 523(7560):337–341 26030524 10.1038/nature14432 PMC4506234

[48] Ma Q (2017) Outflow of cerebrospinal fluid is predominantly through lymphatic vessels and is reduced in aged mice. Nat Commun 8(1):1434 29127332 10.1038/s41467-017-01484-6 PMC5681558

[49] Hickey WF, Hsu BL, Kimura H (1991) T-lymphocyte entry into the central nervous system. J Neurosci Res 28(2):254–260 2033653 10.1002/jnr.490280213

[50] Merlini A (2022) Distinct roles of the meningeal layers in CNS autoimmunity. Nat Neurosci 25(7):887–899 35773544 10.1038/s41593-022-01108-3

[51] Louveau A (2018) CNS lymphatic drainage and neuroinflammation are regulated by meningeal lymphatic vasculature. Nat Neurosci 523:337 10.1038/s41593-018-0227-9 PMC6214619 30224810

[52] Patel N (2009) Venous Anatomy and Imaging of the First Centimeter. Seminars in Ultrasound, CT and MRI 30(6):513–524 10.1053/j.sult.2009.08.003 20099637

[53] Mancini M (2015) Head and neck veins of the mouse. A magnetic resonance, micro computed tomography and high frequency color Doppler ultrasound study. PLoS ONE 10(6):e0129912 26067061 10.1371/journal.pone.0129912 PMC4466257

[54] García-González U (2009) The diploic venous system: surgical anatomy and neurosurgical implications. Neurosurg Focus 27(5):E2 10.3171/2009.8.FOCUS09169 19877793

[55] Toriumi H (2011) Developmental and circulatory profile of the diploic veins. Microvasc Res 81(1):97–102 21093458 10.1016/j.mvr.2010.11.004

[56] Mecheri B, Paris F, Lübbert H (2018) Histological investigations on the dura mater vascular system of mice. Acta Histochemica 120(8):846–857 30292321 10.1016/j.acthis.2018.09.009

[57] Ringstad G, Eide PK (2020) Cerebrospinal fluid tracer efflux to parasagittal dura in humans. Nat Commun 11(1):354 31953399 10.1038/s41467-019-14195-x PMC6969040

[58] Ringstad G, Eide PK (2022) Molecular trans-dural efflux to skull bone marrow in humans with CSF disorders. Brain 145(4):1464–1472 34849609 10.1093/brain/awab388 PMC9128823

[59] Pulous FE (2022) Cerebrospinal fluid can exit into the skull bone marrow and instruct cranial hematopoiesis in mice with bacterial meningitis. Nat Neurosci 25(5):567–576 35501382 10.1038/s41593-022-01060-2 PMC9081225

[60] Upton ML, Weller RO (1985) The morphology of cerebrospinal fluid drainage pathways in human arachnoid granulations. J Neurosurg 63(6):867–875 4056901 10.3171/jns.1985.63.6.0867

[61] Kapoor KG (2008) Cerebrospinal fluid outflow: an evolving perspective. Brain Res Bull 77(6):327–334 18793703 10.1016/j.brainresbull.2008.08.009

[62] Cure JK, Van Tassel P, Smith MT (1994) Normal and variant anatomy of the dural venous sinuses. Semin Ultrasound CT MR 15(6):499–519 7880564 10.1016/s0887-2171(05)80019-8

[63] Tsutsumi S (2014) Cranial arachnoid protrusions and contiguous diploic veins in CSF drainage. Am J Neuroradiol 35(9):1735–1739 24948506 10.3174/ajnr.A4007 PMC7966289

[64] Shah T (2023) Arachnoid granulations are lymphatic conduits that communicate with bone marrow and dura-arachnoid stroma. J Exp Med 220(2):e20220618. 36469302 10.1084/jem.20220618 PMC9728136

[65] Mollgard K (2023) A mesothelium divides the subarachnoid space into functional compartments. Science 379(6627):84–88 36603070 10.1126/science.adc8810

[66] Krisch B (1988) Ultrastructure of the meninges at the site of penetration of veins through the dura mater, with particular reference to Pacchionian granulations. Investigations in the rat and two species of New-World monkeys (Cebus apella, Callitrix jacchus). Cell Tissue Res 251(3):621–31 3130190 10.1007/BF00214011

[67] Liu X (2013) The superficial glia limitans of mouse and monkey brain and spinal cord. Anat Rec (Hoboken, N.J. : 2007) 296(7):995–1007 10.1002/ar.22717 23674345

[68] Hsu M (2019) Neuroinflammation-induced lymphangiogenesis near the cribriform plate contributes to drainage of CNS-derived antigens and immune cells. Nat Commun 10(1):229 30651548 10.1038/s41467-018-08163-0 PMC6335416

[69] Herisson F (2018) Direct vascular channels connect skull bone marrow and the brain surface enabling myeloid cell migration. Nat Neurosci 21(9):1209–1217 30150661 10.1038/s41593-018-0213-2 PMC6148759

[70] Yao H (2018) Leukaemia hijacks a neural mechanism to invade the central nervous system. Nature 560(7716):55–60 30022166 10.1038/s41586-018-0342-5 PMC10257142

[71] Wolfe H (2018) Infiltrating macrophages contribute to age-related neuroinflammation in C57/BL6 mice. Mech Ageing Dev 173:84–91 29758231 10.1016/j.mad.2018.05.003

[72] Batterman KV (2021) T cells actively infiltrate the white matter of the aging monkey brain in relation to increased microglial reactivity and cognitive decline. Front Immunol 12:691 10.3389/fimmu.2021.607691 PMC7920950 33664743

[73] Stichel CC, Luebbert H (2007) Inflammatory processes in the aging mouse brain: participation of dendritic cells and T-cells. Neurobiol Aging 28(10):1507–1521 16959379 10.1016/j.neurobiolaging.2006.07.022

[74] Honarpisheh P, Bryan RM, McCullough LD (2022) Aging Microbiota-gut-brain axis in stroke risk and outcome. Circ Res 130(8):1112–1144 35420913 10.1161/CIRCRESAHA.122.319983 PMC9674376

[75] Yanev P (2019) Impaired meningeal lymphatic vessel development worsens stroke outcome. J Cereb Blood Flow Metab 79:271678X18822921 10.1177/0271678X18822921 PMC7370617 30621519

[76] Esposito E (2019) Brain-to-cervical lymph node signaling after stroke. Nat Commun 10(1):5306 31757960 10.1038/s41467-019-13324-w PMC6876639

[77] Beuker C (2022) Stroke induces disease-specific myeloid cells in the brain parenchyma and pia. Nat Commun 13(1):945 35177618 10.1038/s41467-022-28593-1 PMC8854573

[78] Pu T (2019) Persistent malfunction of glymphatic and meningeal lymphatic drainage in a mouse model of subarachnoid hemorrhage. Exp Neurobiol 28(1):104–118 30853828 10.5607/en.2019.28.1.104 PMC6401547

[79] Rajan WD (2020) Defining molecular identity and fates of CNS-border associated macrophages after ischemic stroke in rodents and humans. Neurobiol Dis 137:104722 31926295 10.1016/j.nbd.2019.104722

[80] Pedragosa J (2018) CNS-border associated macrophages respond to acute ischemic stroke attracting granulocytes and promoting vascular leakage. Acta Neuropathol Commun 6(1):76 30092836 10.1186/s40478-018-0581-6 PMC6083589

[81] Riew TR (2022) Infiltration of meningeal macrophages into the Virchow-Robin space after ischemic stroke in rats: Correlation with activated PDGFR-beta-positive adventitial fibroblasts. Front Mol Neurosci 15:1033271 36644619 10.3389/fnmol.2022.1033271 PMC9837109

[82] Kosel S (1997) Long-lasting perivascular accumulation of major histocompatibility complex class II-positive lipophages in the spinal cord of stroke patients: possible relevance for the immune privilege of the brain. Acta Neuropathol 94(6):532–538 9444354 10.1007/s004010050747 PMC7086531

[83] Xiang J (2017) The choroid plexus as a site of damage in hemorrhagic and ischemic stroke and its role in responding to injury. Fluids Barriers CNS 14(1):8 28351417 10.1186/s12987-017-0056-3 PMC5371201

[84] Li Y, Chen J, Chopp M (2002) Cell proliferation and differentiation from ependymal, subependymal and choroid plexus cells in response to stroke in rats. J Neurol Sci 193(2):137–146 11790394 10.1016/s0022-510x(01)00657-8

[85] Ennis SR, Keep RF (2006) The effects of cerebral ischemia on the rat choroid plexus. J Cereb Blood Flow Metab 26(5):675–683 16136054 10.1038/sj.jcbfm.9600224

[86] Llovera G (2017) The choroid plexus is a key cerebral invasion route for T cells after stroke. Acta Neuropathol 134(6):851–868 28762187 10.1007/s00401-017-1758-y

[87] Ge R (2017) Choroid plexus-cerebrospinal fluid route for monocyte-derived macrophages after stroke. J Neuroinflammation 14(1):153 28754163 10.1186/s12974-017-0909-3 PMC5534106

[88] Rayasam A (2022) Scavenger receptor CD36 governs recruitment of myeloid cells to the blood-CSF barrier after stroke in neonatal mice. J Neuroinflammation 19(1):47 35148760 10.1186/s12974-022-02388-z PMC8840310

[89] van Rooijen N, Sanders A, van den Berg TK (1996) Apoptosis of macrophages induced by liposome-mediated intracellular delivery of clodronate and propamidine. J Immunol Methods 193(1):93–99 8690935 10.1016/0022-1759(96)00056-7

[90] Polfliet MMJ (2001) A method for the selective depletion of perivascular and meningeal macrophages in the central nervous system. J Neuroimmunol 116(2):188–195 11438173 10.1016/s0165-5728(01)00282-x

[91] Wan H (2021) Role of perivascular and meningeal macrophages in outcome following experimental subarachnoid hemorrhage. J Cereb Blood Flow Metab 41(8):1842–1857 33444089 10.1177/0271678X20980296 PMC8327101

[92] Coulibaly AP (2021) Neutrophil enzyme myeloperoxidase modulates neuronal response in a model of subarachnoid hemorrhage by venous injury. Stroke 52(10):3374–3384 34404234 10.1161/STROKEAHA.120.033513 PMC8478903

[93] Drieu A (2020) Alcohol exposure-induced neurovascular inflammatory priming impacts ischemic stroke and is linked with brain perivascular macrophages. JCI Insight 5(4):e129226 31990687 10.1172/jci.insight.129226 PMC7101143

[94] Strbian D (2006) Cerebral mast cells regulate early ischemic brain swelling and neutrophil accumulation. J Cereb Blood Flow Metab 26(5):605–612 16163296 10.1038/sj.jcbfm.9600228

[95] Arac A (2014) Evidence that meningeal mast cells can worsen stroke pathology in mice. Am J Pathol 184(9):2493–2504 25134760 10.1016/j.ajpath.2014.06.003 PMC4188278

[96] McKittrick CM, Lawrence CE, Carswell HV (2015) Mast cells promote blood brain barrier breakdown and neutrophil infiltration in a mouse model of focal cerebral ischemia. J Cereb Blood Flow Metab 35(4):638–647 25564235 10.1038/jcbfm.2014.239 PMC4420882

[97] Strbian D (2007) Mast cell blocking reduces brain edema and hematoma volume and improves outcome after experimental intracerebral hemorrhage. J Cereb Blood Flow Metab 27(4):795–802 16926842 10.1038/sj.jcbfm.9600387

[98] Brea D (2021) Stroke affects intestinal immune cell trafficking to the central nervous system. Brain Behav Immun 96:295–302 33989742 10.1016/j.bbi.2021.05.008 PMC8672365

[99] Garcia JH (1994) Influx of leukocytes and platelets in an evolving brain infarct (Wistar rat). Am J Pathol 144(1):188–199 8291608 PMC1887114

[100] Enzmann G (2013) The neurovascular unit as a selective barrier to polymorphonuclear granulocyte (PMN) infiltration into the brain after ischemic injury. Acta Neuropathol 125(3):395–412 23269317 10.1007/s00401-012-1076-3 PMC3578720

[101] Pérez-de Puig I (2015) Neutrophil recruitment to the brain in mouse and human ischemic stroke. Acta Neuropathol 129(2):239–257 25548073 10.1007/s00401-014-1381-0

[102] Möller K (2014) Sterile Inflammation after Permanent Distal MCA Occlusion in Hypertensive Rats. J Cereb Blood Flow Metab 34(2):307–315 24220169 10.1038/jcbfm.2013.199 PMC3915208

[103] Otxoa-de-Amezaga A (2019) Location of neutrophils in different compartments of the damaged mouse brain after severe ischemia/reperfusion. Stroke 50(6):1548–1557 31084324 10.1161/STROKEAHA.118.023837

[104] Hawkes CA, McLaurin J (2009) Selective targeting of perivascular macrophages for clearance of beta-amyloid in cerebral amyloid angiopathy. Proc Natl Acad Sci U S A 106(4):1261–1266 19164591 10.1073/pnas.0805453106 PMC2633563

[105] Park L (2017) Brain perivascular macrophages initiate the neurovascular dysfunction of Alzheimer Aβ peptides. Circ Res 121(3):258–269 28515043 10.1161/CIRCRESAHA.117.311054 PMC5522360

[106] Rohde D (2022) Bone marrow endothelial dysfunction promotes myeloid cell expansion in cardiovascular disease. Nat Cardiovasc Res 1(1):28–44 35747128 10.1038/s44161-021-00002-8 PMC9216333

[107] Kaiser D (2014) Spontaneous white matter damage, cognitive decline and neuroinflammation in middle-aged hypertensive rats: an animal model of early-stage cerebral small vessel disease. Acta Neuropathol Commun 2(1):169 25519173 10.1186/s40478-014-0169-8 PMC4279586

[108] Iyonaga T (2020) Brain perivascular macrophages contribute to the development of hypertension in stroke-prone spontaneously hypertensive rats via sympathetic activation. Hypertens Res 43(2):99–110 31541222 10.1038/s41440-019-0333-4

[109] Yu Y (2010) Brain perivascular macrophages and the sympathetic response to inflammation in rats after myocardial infarction. Hypertension 55(3):652–659 20142564 10.1161/HYPERTENSIONAHA.109.142836 PMC2890291

[110] Santisteban MM (2020) Endothelium-Macrophage Cross-talk Mediates Blood-Brain Barrier Dysfunction in Hypertension. Hypertension 76(3):795–807 32654560 10.1161/HYPERTENSIONAHA.120.15581 PMC7429290

[111] Faraco G (2016) Perivascular macrophages mediate the neurovascular and cognitive dysfunction associated with hypertension. J Clin Investig 126(12):4674–4689 27841763 10.1172/JCI86950 PMC5127678

[112] Santisteban MM (2022) Meningeal IL-17 producing T cells mediate cognitive impairment in salt-sensitive hypertension. bioRxiv. 10.1101/2022.09.05.506398 PMC10999222 38049579

[113] Hartlehnert M (2021) Bcl6 controls meningeal Th17-B cell interaction in murine neuroinflammation. Proc Natl Acad Sci U S A 118(36):e2023174118 34479995 10.1073/pnas.2023174118 PMC8433502

[114] Bolte AC (2023) The meningeal transcriptional response to traumatic brain injury and aging. Elife 12:e81154 36594818 10.7554/eLife.81154 PMC9810333

